# Identification of drug-related cardiovascular risks: A comprehensive analysis using the FAERS database (2004–2024)

**DOI:** 10.1371/journal.pone.0349218

**Published:** 2026-06-01

**Authors:** Naishen Qin, Yumei Gan, Jun Ouyang, Jiahui Qin, Yuming Tang, Haili Wu, Shumin Wei, Hui Wang, Jiangnan Huang

**Affiliations:** 1 School of Pharmacy, Guangxi Medical University, Nanning, Guangxi, China; 2 Department of Cardiology, The First Affiliated Hospital of Guangxi Medical University, Nanning, Guangxi, China; University Hospital of Padova, ITALY

## Abstract

**Objective:** This study aimed to analyze drug adverse events, identify high-risk medications associated with cardiovascular disease (CVD), and provide evidence for clinical medication safety. **Methods:** We conducted a retrospective analysis of the FAERS database (2004Q1–2024Q4) using nine CVD-related terms from MedDRA 27.0. Four signal detection methods—PRR, ROR, BCPNN, and MGPS—were employed to identify significant drug—event associations. **Results:** A total of 1,560,242 CVD-related reports were included. Rofecoxib, phenylpropanolamine and rosiglitazone were strongly associated with CVD, with ROR values of 73.4 (95% CI: 71.16–75.7), 66.8 (95% CI: 23.25–191.94), and 58.69 (95% CI: 57.68–59.76), respectively. These drugs have since been withdrawn or restricted. Subgroup analysis revealed gender and age differences in CVD risk: testosterone related drugs may be associated with cardiovascular disease risk in elderly and male patients, while alendronic acid may be associated with increased cardiovascular risk in female patients. These preliminary associations may stem from mixed indications rather than direct effects of the drugs. Validate and quantify in the real world doxorubicin and other anticancer agents are correlated with cardiotoxicity in children known risks. Additionally, potential associations with previously unreported CVD risks were suggested for paricalcitol, busulfan, gemtuzumab ozogamicin, clofarabine, and clofazimine, which warrant further attention. **Conclusion:** This study showed that some drugs were significantly associated with CVD, and some of them were newly discovered signals. These findings provide a basis for the generation of new hypotheses about the cardiovascular risk of drugs, but the causal relationship needs to be confirmed by further research.

## Introduction

Cardiovascular disease (CVD) remains the leading cause of mortality and morbidity worldwide and poses a substantial threat to society because of its high prevalence and mortality rates [1]. According to the 2025 American Heart Association report and global data, CVD accounted for 39.5% of total deaths in the United States in 2022, with coronary artery disease being the predominant contributor. The age-adjusted CVD mortality rate has reached 224.3 per 100,000 people [2]. Furthermore, 2020 data from the U.S. Centers for Disease Control and Prevention (CDC) indicated that CVD incurred over 216 billion total healthcare expenditures, along with 147 billion economic losses due to work absenteeism and a decline in productivity [3]. Given this significant disease burden, identifying modifiable drug-related risk factors is critically important.

CVD encompasses a spectrum of disorders affecting the heart and vascular system, with its most severe manifestations including atherosclerotic cardiovascular events such as myocardial infarction, stroke, and peripheral artery disease [4]. The pathophysiology of CVD fundamentally involves the interplay of vascular endothelial dysfunction, inflammatory responses, and thrombogenesis, with oxidative stress also leading to disease pathogenesis [5]. Of particular concern in pharmacotherapy is drug-induced CVD, where anticancer drug-related cardiotoxicity has emerged as a critical issue. With the increasing use of tumor drugs that prolong patient survival, the cardiotoxic effects of anticancer drugs are becoming more prominent [6]. Anthracyclines and related antineoplastic agents exert their toxic effects through mitochondrial ROS generation, altered Ca^2+^ signaling, and subsequent DNA damage in cardiomyocytes, ultimately leading to heart failure and cellular apoptosis [7]. Among classic cardiovascular risk medications, COX-2-selective inhibitors (coxibs) represent a well-documented class associated with serious adverse cardiovascular events, including atherosclerosis, hypertension, myocardial infarction, stroke, heart failure, arrhythmias, and sudden cardiac death. Clinical studies have demonstrated that high-dose NSAID therapy in ankylosing spondylitis patients significantly increases CVD risk, particularly for ischemic heart disease, stroke, and congestive heart failure [8]. These safety concerns have led to voluntary market withdrawal of certain coxibs (e.g., rofecoxib and valdecoxib), whereas celecoxib remains available with an FDA-mandated black box warning for cardiovascular risks. This paradigmatic case revealed deficiencies in pharmacovigilance systems, prompting the scientific community to adopt more comprehensive approaches for data collection and interpretation, thereby advancing evidence-based risk mitigation strategies [9,10]. While the heart is not a common target organ for adverse drug reactions, drug-induced cardiotoxicity remains a significant challenge in pharmaceutical development and clinical practice, with certain medications capable of precipitating severe cardiac events [11].

The FDA Adverse Event Reporting System (FAERS), which is maintained by the U.S. Food and Drug Administration, serves as a comprehensive repository of drug-related adverse events, providing valuable real-world data for investigating drug‒CVD associations [12]. As a critical pharmacovigilance tool, FAERS offers distinct advantages in detecting rare or long-term adverse drug reactions because of its extensive data volume and broad coverage, thereby addressing current research gaps. However, limitations inherent to this spontaneous reporting system, including reporting bias and confounding factors, must be carefully considered. The manuscript uses standard abbreviations throughout and a complete list of abbreviations is provided in S1 Table.

## Materials and methods

### Data sources

FAERS is a publicly available global database maintained by the FDA. The adverse event data in the FAERS are collected primarily through voluntary reports from healthcare professionals, pharmaceutical companies, and the general public. The database is characterized by its large scale and high reliability, with quarterly updates. FAERS includes relevant files such as patient information (DEMO), adverse events (REAC), drug information (DRUG) and event outcomes (OUTC). All the FAERS data can be freely downloaded from https://fis.fda.gov/extensions/FPD-QDE-FAERS/FPD-QDE-FAERS.html. All patient data in this study were obtained from FAERS database, which contains fully de-identified information.

### Study design

In the FAERS database, adverse events (AEs) were coded via Preferred Terms (PTs) on the basis of the Medical Dictionary for Regulatory Activities (MedDRA, version 27.0). All PTs representing symptoms, signs, and potentially related examinations could be categorized into narrative clinically relevant groups via Standardized MedDRA Queries (SMQ). In this study, referring to previous literature [13], CVD can be classified into nine narrow SMQ: embolic and thrombotic events, hypertension, pulmonary hypertension, heart failure, cardiomyopathy, arrhythmias, ischemic heart disease, torsade de pointes/QT prolongation, and noninfectious myocarditis/pericarditis. Additionally, only cases where the drug was listed as the “primary suspect” (PS) for CVD were included.

### Data cleaning

This study extracted American Standard Code for Information Interchange (ASCII) data packages from FAERS(last accessed on March 12, 2025), covering the period from the first quarter of 2004 to the fourth quarter of 2024. All downloaded data were imported into R (Version 4.4.2) for subsequent analysis. To address potential duplicate reports of the same case in the FAERS system, this study employed the FDA-recommended standard deduplication method to increase the reliability of the results. Specifically, if CASEID was identical, the record with the most recent FDA_DT was retained. If both CASEID and FDA_DT were identical, the entry with the higher PRIMARYID was selected [14].

### Statistical analysis

This study employed four methods—ROR, PRR, BCPNN, and MGPS—to jointly detect drug safety signals. Drugs with CVD as their primary indication were excluded to eliminate interference from underlying conditions in adverse reaction assessment, ensuring that the identified signals truly originated from drug effects rather than preexisting diseases. Additionally, the Benjamini-Hochberg procedure was applied to control the false-positive rate. Signal detection was performed via the following pharmacovigilance methods: ① Reporting odds ratio (ROR): A signal was considered positive if the number of reports was ≥ 3 and the 95% confidence interval (CI) lower limit was > 1. A higher ROR value indicates a stronger association.② Proportional reporting ratio (PRR): A signal was deemed positive if PRR ≥ 2 and Chi-square (X²) ≥4. Similarly, larger PRR values suggest stronger associations.③ Bayesian confidence propagation neural network (BCPNN): This method evaluates signals on the basis of the information component (IC). A positive signal was defined as an IC025 (lower 95% CI of the IC) >0, with higher IC values indicating stronger associations. ④ Multi-item Gamma Poisson Shrinker (MGPS): This approach uses the empirical Bayesian geometric mean (EBGM) to assess associations. A signal was considered positive if EBGM05 (lower 95% CI of EBGM) was > 2. These Bayesian methods enhance robustness, particularly in handling data sparsity and reporting heterogeneity. To improve reliability, we selected only CVD-associated drugs that met the signal criteria of all four methods simultaneously. Additionally, the Benjamini-Hochberg procedure was applied to raw *p* values (denoted as *p*_adjust) to control the false discovery rate (FDR) [15].

**Fig 1:**
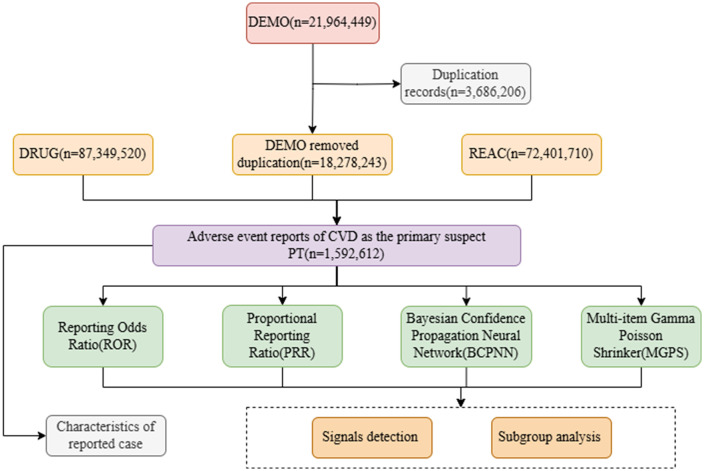
Overall study flowchart. This flowchart illustrates the steps for identifying and evaluating potential drug safety signals related to CVD from the FAERS database.

Statistical visualization was performed via GraphPad Prism 9.5.1 (GraphPad Software, Inc.) and R 4.4.2 (R Core Team) with the ggplot2 package to generate baseline characteristic plots and forest plots. The overall study flowchart (Fig 1) was created via draw.io (version 26.2.2).

## Results

### Baseline characteristics

After data cleaning and organization of the ADE reports from the first quarter of 2004 to the fourth quarter of 2024, a total of 1,560,242 reports involving CVD were screened. The baseline characteristics of CVD patients are presented in Table 1. Among these reports, females accounted for 771,770 cases (49.5%), males accounted for 630,995 cases (40.4%), and 157,477 cases (10.1%) had missing sex information. The age distribution revealed that patients aged 18–64.9 years accounted for the highest proportion, with 542,307 cases (34.8%), followed by those aged 65–85 years, with 423,836 cases (27.2%).

**Table 1:** Baseline characteristics of patients with CVD.

Characteristics	Drug-related CVD (N = 1,560,242)
**Gender**	
Female	771,770 (49.5%)
Male	630,995 (40.4%)
Missing	157,477 (10.1%)
**Weight**	
<50 kg	36,404 (2.3%)
>100 kg	69,554 (4.5%)
50~100 kg	348,924 (22.4%)
Missing	1,105,360 (70.8%)
**Age**	
<18 age	32,121 (2.1%)
>85 age	40,872 (2.6%)
18~64.9 age	542,307 (34.8%)
65~85 age	423,836 (27.2%)
Missing	521,106 (33.4%)
**Occupation**	
Consumer	558,031 (35.8%)
Healthcare Professional	138,263(8.9%)
Lawyer	47,346 (3.0%)
Doctor of Medicine	445,053 (28.5%)
Missing	82,792 (5.3%)
Other health professionals	199,123 (12.8%)
Pharmacist	88,723 (5.7%)
Registered Nurse	910 (0.1%)
**Reporter Country**	
United States	790,857(50.7%)
Canada	99,876(6.4%)
Japan	74,927(4.8%)
France	62,083(4.0%)
Germany	61,636(4.0%)
Others	470,863(30.2%)

**Fig 2:**
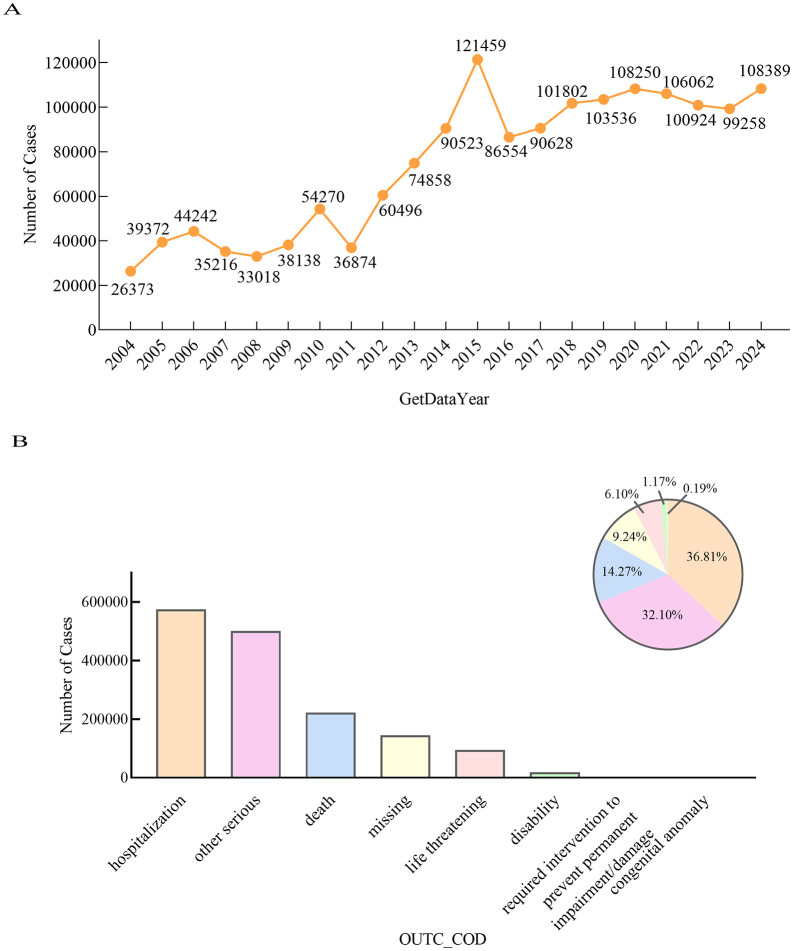
Number of adverse event reports and patient outcomes included in the analysis. A: The number of adverse event reports submitted to FAERS annually from 2004 to 2024. The data shows a fluctuating trend, reaching its peak in 2015; B: This is a distribution figure of patient outcomes. This figure shows that long-term hospitalization is the most common reported outcome.

Among the reporting countries, the United States reported the highest number of CVD cases (790,857), followed by Canada, Japan, France, and Germany (99,876; 74,927; 62,083; and 61,636 cases, respectively). Since the database was established in 2004, there have been annual reports of adverse events related to CVD. The number of reported cases showed a fluctuating upward trend, peaking in 2015 and then entered a renewed phase of annual increase. (Fig 2A). The most common adverse outcome was prolonged hospitalization (574,360 patients) (Fig 2B).

This table summarizes the demographic distribution of 1,560,242 patients in the study cohort, including Gender, Weight, Age, Occupation, and Reporter Country. The data are presented in the form of case numbers and percentages (%).

### Signal detection analysis

A total of 89 drugs had positive signal values, the top five drugs ranked according to ROR are as follows (Table 2) ROR: rofecoxib (n = 31,196; ROR = 73.4; PRR = 10.36; X^2^ = 282,842.83; IC = 3.35; EBGM = 10.18; *p*_adjust = 0), rosiglitazone (n = 74,478; ROR = 58.69; PRR = 10.25; X^2^ = 647,840.78; IC = 3.29; EBGM = 9.81; *p*_adjust = 0), testosterone (n = 12,431; ROR = 5.2; PRR = 3.83; X^2^ = 28,232.26; IC = 1.93; EBGM = 3.81; *p*_adjust = 0), rivaroxaban (n = 29,900; ROR = 3.54; PRR = 10.36; X^2^ = 282,842.83; IC = 3.35; EBGM = 10.18; *p*_adjust = 0), apixaban (n = 29,751; ROR = 3.01; PRR = 2. Here, we list several high signal drugs of known high-risk drugs. The analysis strongly shows that there is a significant correlation between rofecoxib (ROR:73.4), rosiglitazone (ROR:58.69) and cardiovascular risk, which is highly consistent with the known fact of global delisting due to cardiovascular toxicity, indirectly verifying the reliability of the data mining method used in this study. A complete list of all detected signals is provided in S2 Table.

**Table 2:** Signal strength of CVD at the preferred term level in the FAERS database.

DRUG	Case Reports	ROR(95% CI)	PRR(X^2^)	IC(IC025)	EBGM(EBGM05)	p_adjust
rofecoxib	31,196	73.4 (71.16 - 75.7)	10.36 (282,842.83)	3.35 (3.32)	10.18 (9.92)	0
rosiglitazone	74,478	58.69 (57.64 - 59.76)	10.25 (647,840.78)	3.29 (3.28)	9.81 (9.67)	0
testosterone	12,431	5.2 (5.09 - 5.31)	3.83 (28,232.26)	1.93 (1.9)	3.81 (3.74)	0
rivaroxaban	29,900	3.54 (3.49 - 3.58)	2.91 (40,281.25)	1.52 (1.51)	2.88 (2.84)	0
apixaban	29,751	3.01 (2.97 - 3.05)	2.57 (30,708.46)	1.35 (1.33)	2.54 (2.52)	0

This table provides ROR value ranking, because in pharmacovigilance, the lower limit of the 95% confidence interval of ROR is usually more important, which provides a conservative estimate of the correlation strength. If the lower limit of the confidence interval is still greater than 1, the signal is more robust. The common purpose of using four algorithms (ROR, PRR, BCPNN, EBGM) is to quantify the deviation between the reporting proportion of target drug adverse event combination and the background reporting proportion. The p_adjust value is the p value corrected by the Benjamin-Hochberg FDR error detection rate, which is used to control the false positive rate in the multiple hypothesis test. A value of 0 indicates that the association is statistically significant.

### Subgroup analysis

**Fig 3:**
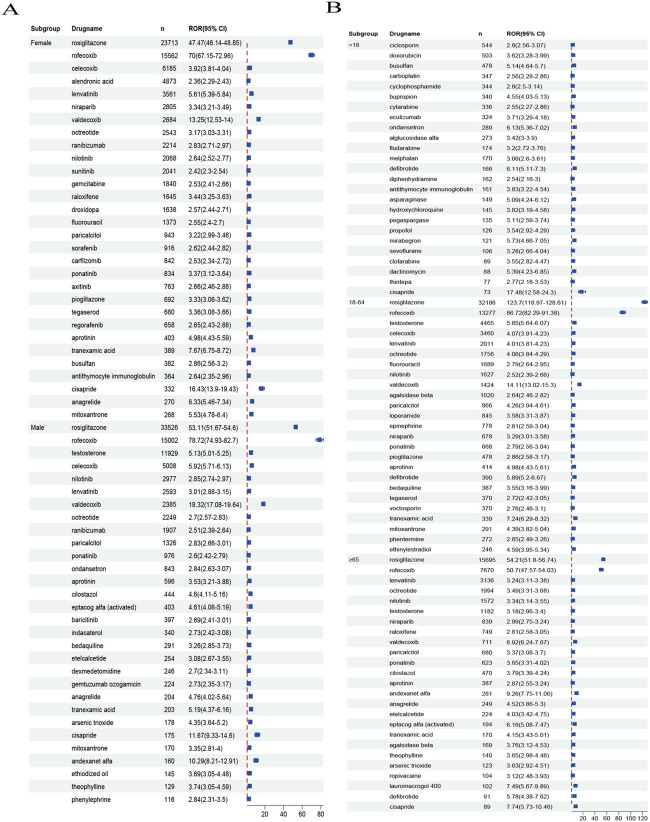
Top-ranked drugs associated with CVD in subgroup analyses. A: Top 30 drugs by reported CVD case numbers in sex subgroups; This figure lists the top 30 drugs with reported cases in both male and female patients. By comparing the drug lists on both sides, it can be visually observed which drugs have significantly higher reported rates in specific gender groups. B: Top 25 drugs by reported CVD case numbers in age subgroups. This figure displays the top 25 drugs in terms of reported cases among patients of different age groups (e.g., < 18 years, 18-64 years, > 64 years). This helps to reveal the age-related medication patterns associated with CVD risk.

In the sex subgroup analysis, female patients most frequently reported receiving rosiglitazone (n = 23,713; ROR = 47.47, 95% CI: 46.14–48.85), rofecoxib (n = 15,682; ROR = 70.97, 95% CI: 67.15–72.96), celecoxib (n = 6,185; ROR = 3.92, 95% CI: 3.81–4.04), alendronic acid (n = 4,873; ROR = 2.36, 95% CI: 2.29–2.43), and lenvatinib (n = 3,561; ROR = 5.61, 95% CI: 5.39–5.84), with rosiglitazone and rofecoxib demonstrating significantly greater signal strengths than other drugs. Among male patients, the top reported drugs were rosiglitazone (n = 33,526; ROR = 53.11, 95% CI:51.67–54.60), rofecoxib (n = 15,002; ROR = 78.72, 95% CI:74.93–82.70), testosterone (n = 11,929; ROR = 5.13, 95% CI:5.01–5.25), celecoxib (n = 5,008; ROR = 5.92, 95% CI:5.71–6.13), and nilotinib (n = 2,977; ROR = 2.85, 95% CI:2.74–2.97), whereas rosiglitazone and rofecoxib again presented the highest ROR values, suggesting potential gender-specific adverse event risks requiring clinical attention (Fig 3A).

In the age subgroup analyses, minors (<18) most commonly reported ciclosporin (544 cases), doxorubicin (503 cases), busulfan (478 cases), carboplatin (347 cases), and cyclophosphamide (344 cases), with cisapride exhibiting a notably high signal value (n = 73; ROR = 17.48, 95% CI: 12.58–24.30). Adults (18–64 years) predominantly reported the use of rosiglitazone (32,186 cases), rofecoxib (13,277 cases), testosterone (4,465 cases), celecoxib (3,460 cases), lenvatinib (2,011 cases), and octreotide (1,756 cases), whereas elderly patients (≥65) most frequently reported the use of rosiglitazone (15,695 cases), rofecoxib (7,670 cases), lenvatinib (3,136 cases), octreotide (1,994 cases), nilotinib (1,572 cases), and testosterone (1,182 cases), with rosiglitazone and rofecoxib consistently showing the highest ROR values across all age groups (Fig 3B).

## Discussion

CVD, as one of the leading causes of mortality and disability worldwide, has drawn significant attention because of its association with drug safety [16]. Our study conducted a 20-year analysis of the FAERS database, representing, to our knowledge, the most comprehensive investigation to date in terms of both scope and depth for identifying drug-associated CVD risks via this database, thereby providing scientific evidence for CVD risk reduction and rational drug use. We systematically identified 89 drugs significantly associated with CVD risk, among which rosiglitazone, rofecoxib, and testosterone exhibited particularly prominent reporting frequencies and signal strengths (ROR/PRR). The results revealed a greater proportion of female patients (49.5%) than male patients, with the 18–65 age group being the most frequently affected (35.0%), which is consistent with previous studies indicating a higher CVD incidence in middle-aged populations [17]. Subgroup analyses revealed critical population-specific patterns: rosiglitazone (ROR = 53.11) and rofecoxib (ROR = 78.72) showed stronger risk signals in males than in females, potentially attributable to their distinct pharmacological mechanisms and metabolic profiles, which aligns with existing research [18]. Notably, the prokinetic agent cisapride demonstrated an exceptionally high ROR (17.48) in minors, corroborating earlier reports of its association with cardiovascular risks, particularly arrhythmogenic cardiotoxicity [19]. Compared with existing evidence, our study not only validated safety signals of known high-risk drugs (e.g., rofecoxib [ROR = 73.4], which is consistent with the findings of the VIGOR trial [20], but also identified emerging signals requiring vigilance, such as testosterone’s significant cardiovascular risk (ROR = 5.2). However, the sex-specific mechanisms underlying these associations warrant further investigation.

From a pharmacological classification perspective, drugs from different therapeutic categories exhibited distinct risk patterns: antineoplastic agents, including nilotinib, fluorouracil, mitoxantrone, and ponatinib, demonstrated positive associations with CVD. These findings align with established adverse effects such as coronary artery spasm, thrombosis, myocardial ischemia, heart failure, cardiomyopathy, and QTc prolongation [21–24]. Notably, lenvatinib and sorafenib predominantly induce hypertension as their most common grade-level adverse events, which are particularly prominent in both younger and older patients, warranting enhanced cardiac monitoring during treatment [25]. As the first-line therapy for radioactive iodine-refractory differentiated thyroid carcinoma, lenvatinib has received limited attention to its cardiovascular effects. Its antitumor mechanism involves the inhibition of vascular endothelial growth factor receptor (VEGFR) and other targets. However, VEGFR suppression reduces vasodilatory factors, activates the renin‒angiotensin system, and induces endothelial dysfunction, collectively contributing to hypertension, with an incidence rate reaching 68% [26]. A Bayesian network meta-analysis of randomized controlled trials evaluating nine VEGFR tyrosine kinase inhibitors identified lenvatinib and vandetanib as having the most severe cardiotoxic profiles [27]. Our study further suggests their potential association with CVD risk, emphasizing the necessity for cardiovascular health surveillance during clinical use.

Endocrine and metabolic drugs such as the thiazolidinedione rosiglitazone demonstrated a prominent positive signal for cardiovascular risk (n = 74,478; ROR = 58.69). As an antidiabetic agent for type 2 diabetes mellitus, rosiglitazone may increase atherogenic lipoprotein levels in both the fasting and postprandial states. Notably, it increases LDL-C concentrations while maintaining HDL-C ratios and has neutral effects on triglycerides, a profile potentially contributing to cardiovascular risk [28]. Researchers investigating the cardiovascular effects of rosiglitazone through shared data updates have employed multiple statistical sensitivity analyses (e.g., alternate modeling approaches, covariate adjustments, and varying endpoint definitions). These analyses consistently confirmed the robustness of the association of rosiglitazone with increased cardiovascular risk and mortality, with no substantive changes observed across methodological variations [29]. Our study further substantiates these findings, demonstrating the significant positive correlation of rosiglitazone with CVD events, particularly among male and adult populations.

Anti-inflammatory and analgesic agents the selective inhibition of cyclooxygenase-2 (COX-2) induces thromboxane/prostacyclin imbalance, leading to adverse cardiovascular effects. Rofecoxib, celecoxib, and valdecoxib—COX-2 inhibitors primarily used for osteoarthritis and rheumatoid arthritis management—demonstrate significant cardiovascular hazards, including atherosclerosis, hypertension, myocardial infarction, stroke, heart failure, arrhythmias, and sudden cardiac death. The cardiovascular risks of rofecoxib, a selective COX-2 inhibitor, were conclusively established by the VIGOR study [30]. Both rofecoxib and celecoxib were subsequently withdrawn from markets owing to their increased risks of heart attack and stroke, with the FDA additionally recommending voluntary discontinuation of valdecoxib [9].

Gender and age differences in adverse drug events remain critical pharmacovigilance focuses, with studies suggesting that sex-specific drug response variations are mediated by pharmacokinetic (e.g., absorption, distribution, metabolism, excretion), pharmacodynamic, and hormonal factors [31]. This is particularly evident in CVD, which, despite being the leading cause of female mortality, has racial/ethnic disparities [32]. The preliminary analysis of this study shows that patients who use alendronic acid have an increased risk of cardiovascular events (n = 4,873, ROR = 2.36), with a higher number of cases, suggesting the possibility of non accidental phenomena that deserve attention. Based on a thorough analysis of existing literature, we believe that it is more likely to reflect a higher baseline cardiovascular risk among users of alendronate sodium, rather than a direct causal adverse effect of the drug.

In clinical practice, patients prescribed alendronate sodium often suffer from more severe osteoporosis. The multiple risk factors for osteoporotic fractures, such as long-term use of glucocorticoids, anticoagulants, anticonvulsants, aromatase inhibitors, chemotherapy drugs, and gonadotropin-releasing hormone agonists [33], are themselves independent strong risk predictors of cardiovascular disease. Therefore, the observed increased risk is likely due to the inherent high-risk baseline characteristics of the patient population, with alendronate serving more as a ‘marker of disease severity’. A retrospective cohort study by Pittman et al. reported that the use of bisphosphonates is associated with an increased risk of myocardial infarction (hazard ratio 1.38; 95% confidence interval 1.08-1.77; P=0.01) [34]. Similarly, the study by Wang et al. found that the incidence of acute ischemic stroke, atrial fibrillation, and congestive heart failure in patients with osteoporosis was significantly higher than in patients who did not use nitrogen bisphosphonates [35]. However, these findings seem contradictory to some studies reporting that alendronate has cardiovascular neutral or even protective effects [36], as well as Sing et al.’s proposal in a large retrospective analysis in Hong Kong that alendronate is associated with significantly reduced cardiovascular mortality and 1-year myocardial infarction rates. This phenomenon persists in the long term, but at lower levels, and the risk of stroke is slightly lower at 5 and 10 years after hip fracture [37]. More importantly, this difference highlights the importance of adequately adjusting for confounding factors in observational studies. Sing et al.’s study may effectively balance the baseline characteristics of the treated and untreated groups by applying strict, time-dependent propensity score matching, thereby minimizing “indication confounding”-that is, patients with more severe conditions are more likely to be biased by prescription drugs. Pittman et al. did not use propensity score matching and mainly relied on statistical models for adjustment, which may not fully control for the aforementioned biases. Although Wang et al. used a 1:4 age, gender, and comorbidity matching, the number of matching variables was relatively small compared to Sing et al., which may result in residual confounding. Although our study has adjusted for multiple known confounding factors, the possibility of residual confounding cannot be completely ruled out.

From a biological mechanism perspective, bisphosphonates (especially nitrogen-containing compounds such as alendronic acid) have been shown to inhibit vascular calcification and have anti-inflammatory properties [38], providing a plausible biological basis for their cardiovascular neutrality or even benefit. However, this stands in contrast to our preliminary findings. Therefore, we speculate that the preliminary observed association between alendronate sodium and cardiovascular event risk in this study is more likely driven by residual confounding factors that are difficult to fully measure, such as the severity of osteoporosis, complexity of concomitant medications, and overall disease burden, rather than representing a causal relationship. This possibility suggest that clinicians should be more cautious in assessing the inherent baseline cardiovascular risk and strengthening monitoring and management when prescribing alendronate to patients with osteoporosis, especially those with multiple cardiovascular risk factors. Ultimately, elucidating the net effects of alendronate sodium on the cardiovascular system requires further validation through prospective randomized controlled trials targeting specific populations or more precise epidemiological studies that can better control confounding.

Testosterone treatment (n = 11,929; ROR = 5.13) is significantly associated with CVD risk, this finding that is not entirely consistent with the meta-analysis conclusions of most large randomized controlled trials between 2013 and 2020, which generally indicate that testosterone treatment does not significantly increase cardiovascular risk [39–42]. Loo et al. (2019) reported contrary findings: analyzing a cohort of men with no evidence of Hypothalamic pituitary thyroid axis disease via the UK Clinical Practice Research Datalink, they associated use of testosterone with higher risk of a composite outcome of stroke, transient ischemic attack or MI, with the risk highest in the first six to 24 months of testosterone replacement therapy use [43]. As stated in Yeap BB et al.‘s review, considering the limitations of these retrospective, observational, and non randomized studies, it is not possible to prove causal relationships [44]. Therefore, the strong risk signals identified in this study should be considered as an association rather than a cause and effect, and testosterone prescriptions may not be fully targeted at appropriate patients. There may be room for optimization in treatment management, such as dose adjustment and level monitoring. Nevertheless, the optimal dosing and long-term effects of testosterone replacement therapy require further investigation.

The age-stratified subgroup analysis revealed distinct drug-induced risk stratification patterns, with immunosuppressants such as ciclosporin (n = 544) and antineoplastic agents such as doxorubicin (n = 503) and cyclophosphamide demonstrating high reporting frequencies among minors, which is consistent with the literature documenting ciclosporin-associated hypertension, doxorubicin/cyclophosphamide-induced cardiomyopathy and heart failure [45–47]. This study further validated and quantified the association between doxorubicin and other anticancer drugs and the known risk of pediatric cardiac toxicity in the real world, and emphasized the necessity of strengthening the monitoring of pediatric cancer drugs. The notably elevated ROR value of cisapride (17.48) warrants particular vigilance, as this prokinetic agent, although withdrawn in multiple countries owing to QT prolongation risks, remains utilized in pediatric gastrointestinal therapy in some regions [48,49]. We should enhance global regulatory coordination to improve drug safety for children. In adult and elderly subgroups, rosiglitazone and rofecoxib consistently dominated adverse event reports, whereas lenvatinib demonstrated significantly higher case numbers in elderly patients (n = 3,136 vs adults: n = 2,011), reflecting both its therapeutic indications for thyroid/liver cancers in older populations and the cardiovascular vulnerability of elderly patients to multitarget tyrosine kinase inhibitors [50].

This study provides three key clinical contributions. First, it recommends reevaluating the risk‒benefit ratios of controversial medications such as rosiglitazone in specific populations (e.g., elderly diabetic patients). Second, it highlights the need for targeted monitoring protocols for emerging risk signals such as testosterone replacement therapy. Third, Validate and quantify in the real world doxorubicin and other anticancer agents are correlated with cardiotoxicity in children known risks, optimized cardioprotective strategies for pediatric anticancer drugs such as doxorubicin are needed. In addition, our analysis revealed significant CVD associations across multiple drug classes, including antineoplastic agents, endocrine/metabolic drugs, and anti-inflammatory/analgesic medications. Notably, testosterone may demonstrate positive correlations in male and elderly patients—a finding that is consistent with the male-specific risks documented in DailyMed drug labels (https://dailymed.nlm.nih.gov/dailymed/). Additionally, we uncovered previously unreported drug‒CVD associations for paricalcitol, busulfan, gemtuzumab ozogamicin, clofarabine, and clofazimine, which are not currently addressed in prescribing information.

The results of this study have important implications for clinical practice and pharmacovigilance. First of all, our analysis successfully reproduced the powerful ability of the faers database to capture the cardiovascular risk of withdrawn drugs (such as rofecoxib), which verified the effectiveness of drug safety monitoring using such real-world data sources. Secondly, although the causal relationship of controversial signals (such as testosterone) in the study is not yet clear, the study itself provides important warnings for clinicians. It is suggested that patients with specific cardiovascular risk factors should be more strictly evaluated and monitored when prescribing. From a broader perspective, this study shows that spontaneous reporting systems such as faers are indispensable tools to ensure drug safety and improve the quality of medical services. Through continuous data accumulation and analysis, we can provide key evidence for regulatory decision-making, so as to continuously optimize clinical practice and ultimately maximize the protection of patients’ interests. However, this study recognized the inherent limitations of the faers database, such as reporting bias and uncontrollable confounding factors (such as patient complications and drug combination). Due to the lack of complete patient medical record details in the database, we cannot statistically adjust these confounding factors (such as hierarchical analysis or multivariate model). Therefore, the signals detected in this study should be regarded as correlation signals rather than causal evidence. These signals provide important assumptions for potential drug risks, but need to be further verified by actual research or clinical trials.

## Conclusion

This study highlights the need for enhanced pharmacovigilance and optimized therapeutic strategies, as multiple pharmacological classes may be associated with CVD. Additionally, all findings should be substantiated through additional clinical investigations and experimental studies.

## Supporting information

S1 Table List of abbreviations used in the study. This table provides the full terms and corresponding abbreviations for key concepts, databases, statistical methods, and medical terms mentioned throughout the manuscript.
(XLSX)

S2 Table Complete list of all detected drug-adverse event signals. This table provides the exhaustive results of the disproportionality analysis, including all four statistical metrics (ROR, PRR, BCPNN, MGPS) for each drug-event pair.
(XLSX)
